# The Role of miRNAs in the Resistance of Anthracyclines in Breast Cancer: A Systematic Review

**DOI:** 10.3389/fonc.2022.899145

**Published:** 2022-05-12

**Authors:** Zihan Si, Yan Zhong, Sixian Lao, Yufeng Wu, Guoping Zhong, Weiwei Zeng

**Affiliations:** ^1^ Institute of Clinical Pharmacology, School of Pharmaceutical Sciences, Sun Yat-sen University, Guangzhou, China; ^2^ The Second People's Hospital of Longgang District, Shenzhen, China; ^3^ Shenzhen Baoan Women’s and Children’s Hospital, Jinan University, Shenzhen, China; ^4^ Guangdong Provincial Key Laboratory of New Drug Design and Evaluation, Guangzhou, China

**Keywords:** breast cancer, miRNAs, anthracyclines, drug resistance, efficacy

## Abstract

Breast cancer has been reported as the most common cancer in women globally, with 2.26 million new cases in 2020. While anthracyclines are the first-line drug for breast cancer, they cause a variety of adverse reactions and drug resistance, especially for triple-negative breast cancer, which can lead to poor prognosis, high relapse, and mortality rate. MicroRNAs (miRNAs) have been shown to be important in the initiation, development and metastasis of malignancies and their abnormal transcription levels may influence the efficacy of anthracyclines by participating in the pathologic mechanisms of breast cancer. Therefore, it is essential to understand the exact role of miRNAs in the treatment of breast cancer with anthracyclines. In this review, we outline the mechanisms and signaling pathways involved in miRNAs in the treatment of breast cancer using anthracyclines. The role of miRNA in the diagnosis, prognosis and treatment of breast cancer patients is discussed, along with the involvement of miRNAs in chemotherapy for breast cancer.

## Introduction

Different kinds of treatments are applied to different immunohistological types of breast cancer. Triple-negative breast cancer (TNBC), which is not sensitive to endocrine and targeted therapies, is treated with a combination of anthracyclines in the clinical. As the first-line drug class for TNBC, anthracyclines can influence the development of tumors by inhibiting the synthesis of biological DeoxyriboNucleic Acid(DNA). Studies have shown that anthracyclines can affect the activity of DNA topoisomerase to stabilize the DNA double-strand and prevent DNA replication (1, 2). Since daunorubicin, the first anthracycline drug, was discovered, many other anthracycline drugs have been developed one after another, including adriamycin, epirubicin, clarithromycin, and so on. They have been widely used in the treatment of hematological malignancies and solid tumors. However, the frequent use of anthracyclines can cause drug resistance to develop gradually. As a result, cancer will continue to develop, proliferate, invade and metastasize, and eventually lead to patient death. Meanwhile, many adverse reactions caused by anthracyclines, in particular, cardiotoxicity, also affect the prognosis of patients. Clinical studies have shown that this adverse reaction is often irreversible and fatal to patients. Even the first use of anthracyclines may cause heart damage (3). In addition, it can also induce liver damage (4) and promote breast cancer metastasis to the lungs (5). Therefore, many researchers are now working on the mechanisms of breast cancer cell resistance to anthracyclines and the mechanisms of anthracycline adverse reactions.

As a non-coding Ribonucleic Acid(RNA), miRNA is composed of approximately 20 nucleotides, and participates in the regulation of RNA transcription by activating or blocking the translation of mRNA targets. More and more studies have found that miRNA plays a significant role in biological processes, such as embryonic development, cell proliferation, differentiation, migration, apoptosis, and signal transduction (6). Therefore, the abnormal expression of miRNA is closely related to the development of tumor cells (7). In recent years, many studies have shown that miRNA is involved in the regulation of the efficacy of anthracyclines in the treatment of breast cancer, and may be used as a specific biomarker for diagnosis, control and prediction of adverse reactions. Through reviewing past research, this article summarizes the effects of various miRNAs in breast cancer treated by anthracyclines, and provides certain guidance for future research. At the same time, we prove the clinical application potential of various miRNAs in risk prediction, diagnosis and treatment.

## MiRNAs and Anthracyclines Resistance

An important problem to overcome in the application of anthracyclines is drug resistance. The emergence of drug resistance makes tumor cells more aggressive and metastatic and leads to failure in clinical treatments, eventually leading to patient death (8). Therefore, in hopes of overcoming the resistance of anthracyclines through miRNA regulation, scientists have been studying the mechanism of drug resistance. To modify sensitization, transformation, and metastasis, miRNAs regulate their downstream signaling pathways or associated proteins by modulating their target genes. Increased drug efflux *via* altering cell membrane transporters is one of the mechanisms of drug resistance generated by anthracyclines, according to Li, X.J. et al.The key factor is the ATP-bindingcassette transporter (ABC) family and its derivatives, P-glycoprotein (P-gp), Multidrug Resistance-associated Protein (MRP) and topoisomerase II. The other potential mechanism of drug resistance is the inhibition of tumor cell apoptosis. Anthracycline reduces tumor cell apoptosis and increases cell survival and causes cell cycle arrest, then activates the epithelial-mesenchymal transition (EMT) by activating or inhibiting cell signaling transduction pathways (9). In addition, the drug resistance of tumors depends on some tumor proteins, which can affect the metabolic function of tumor cells and control their microenvironment.

Wang, Y.D. et al. reported that the target genes of miRNAs can be divided into four types: oncogenes, tumor suppressor genes, signaling pathway genes, and cell cycle regulatory genes (10). The upstream or downstream of these pathways are also interspersed with various apoptotic proteins, oncogenes, and tumor suppressor genes to affect cell apoptosis, changes in EMT processes, and cell cycle arrest. Accumulating studies have found that one miRNA may be able to regulate multiple genes, and these genes can cooperate to influence each other, and even produce feedback loops.

## MiRNAs and Membrane Transport

The factors that cause changes in cell membrane transport mainly include the ABC family, P-gp protein, MRP protein, topoisomerase, etc. MiRNAs participate in their regulation cells to anthracycline drugs. Xie, M.X. et al. demonstrated that the expression of miR-132 and miR-212 inhibits the expression of nuclear factor kappa-β (NF-κβ), which leads to an increase in the expression of breast cancer resistance protein (BCRP), a member of ABC family, and reduces the sensitivity of tumor cells to anthracyclines (11). Some studies have reported that miR-128 exerts its function of reducing drug resistance of tumor cells by inhibiting the B-cell- specific Moloney murine leukemia virusintegration site-1 (Bmi-1) and ABCC5 (9, 12, 13). The relationship between miR-760 and ABCA1 has also been studied. The high expression of miR-760 can mediate the decline of tumor cell resistance to drugs by reducing the expression of ABCA1 (13). There was a negative correlation between miR-134 and ABCC1, the high expression of miR-134 resulting in reduced tumor cell proliferation and increased apoptosis, which reflects its potential to reduce drug resistance (14). miR-145, miR-451, miR-326, and miR-199a are also implicated in MRP1 (ABCC1) (15–18). Di, H. et al. reported that miR-124-3p enhanced the sensitivity of tumor cells to drugs by reducing the expression of ABCC4 (19).

P-gp is a molecular pump located on the cell membrane to protect cells from harmful exogenous molecules (20). However, this protective mechanism on tumor cells can hinder the entry of anticancer drugs, thereby inducing drug resistance. P-gp genes can be downstream targets of many oncogenes and tumor suppressor genes. MiRNAs affect the number of transcription and synthesis of P-gp through modulating related signaling pathways. Research showed that miR-302s can cause cancer cells susceptible to anthracyclines by down-regulating the expression of P-gp through the MAP/ERK kinase 1 (MAKK1) pathway (21). The overexpression of miR-195 can inhibit the expression of P-gp through the Raf-1 signaling pathway, making more breast cancer cells susceptible to apoptosis and increasing their sensitivity to anthracyclines (22).

### MiRNAs and Cell Apoptosis

Most of the miRNAs achieve their aims by regulating signaling pathways. The most important and widely studied pathways are the Phosphatase and tensin homolog deleted on chromosome ten- phosphatidylinositol 3 kinase- AKT (PTEN-PI3K-AKT) signaling pathway, Notch signaling pathway, Ras signaling p–athway, etc (23). The antiapoptotic protein, B cell leukemia oncogene (Bcl) protein family is the most important link in cell apoptosis, including Bcl-2, Bcl-Xl, Mcl-1, Bcl-w, and so on (24). Many studies have shown that various signaling pathways related to apoptosis have a common junction, which is regulated by the Bcl family. Therefore, Bcl family proteins can inhibit cell death caused by a variety of cytotoxic factors. This phenomenon is even more prominent in tumor cells. Many researchers are trying to link miRNAs with the expression of Bcl family proteins to find a therapeutic strategy to reduce the resistance of tumor cells to anthracyclines. miR-181a is a well recognized miRNA at present. Ying, Z. et al. found that Bcl-2 was decreased by increasing the expression of miR-181a and induced the apoptosis of mitochondria, thereby increasing the apoptosis induced by doxorubicin drugs (25). There is evidence that the overexpression of miR-192-5p will also reduce the expression of Bcl-2, which makes the Bcl-2-Asociated Agonist of Cell Death (BAD) gene competitively increases. As a result, the expression of Peptidylprolyl Isomerase A (PPIA) is inhibited and the resistance of tumor cells to anthracyclines reduced (26). Furthermore, miR-122-5p, miR-195, miR-125b, miR-193b, miR-34a, and miR-200c are negatively related to the regulation of Bcl-2 or Myeloid cell leukemia sequence (Mcl) family and other Bcl family proteins (24, 27–31). Other studies showed that some miRNAs, such as miR-222, miR-19a, and miR-21, are positively correlated with the expression of the Bcl family (32, 33).

The Bcl activating gene proteins are tumor suppressors. Their mutations can affect the process of cell proliferation, transcription and apoptosis. The relationship between the mutation of P53 and miRNA is attractive to researchers. miR-214 has been shown to down-regulate RFWD2-p53 cascade, causing tumor cells sensitive to anthracyclines (34). Yuan, Y. et al. identified that miR-133a improves sensitization of tumor cells by suppressing the expression of the uncoupling protein-2 (UCP-2) (35). This mechanism is considered to be related to P53 mutation (36). Furthermore, it is interesting to note that miR-191-5p declines the expression of P53, which binds to the promoter of miR-191-5p, forming a negative feedback regulation chain by downregulating the expression of SOX4 (37). Not only can miRNAs change drug resistance by regulating the mutation of P53, recent studies demonstrated that some miRNAs can also be regulated by P53. Lin, S. et al. found that the expression level of miR-30c is controlled by P53 mutation, which could contribute to tumor cell sensitivity to doxorubicin by regulating the Fanconi anemia complementation group F protein (FANCY) and REV1 protein (38). In addition, miR-127, miR-34a, and miR-542-3p are all mentioned to be related to P53 network in other studies (16, 39).

PI3K-AKT is also a classic signaling pathway that can induce the apoptosis of cells. The abnormal expression of PTEN protein is enough to antagonize PI3K-AKT *via* dephosphorylating the Phosphatidylinositol (3–5) Trisphosphate (PIP3) on the cell membrane to produce phosphati-dylinositol-4,5-bisphosphate (PIP2), which plays a role in the growth, apoptosis, adhesion (40), infiltration and migration of cancer cells (41). Once the PI3K-AKT signaling pathway is inhibited or damaged, it will promote the activation of tumor cell apoptosis and reduce the occurrence of drug resistance. It was reported that the expression of miR-222 (42, 43) and miR-29a (44) were decreased after treatment with anthracyclines. As a result, it led to anthracycline resistance because of the decreased of PTEN. In addition, miR-221 has been shown to make the PI3K-AKT pathway more active by targeting the PTEN protein, which strengthens the tolerance of tumor cells to anthracyclines. Many miRNAs, such as miR-21 (45, 46), miR-19a (47), miR-132 (11), and miR-212 (11), are negatively correlated with PTEN expression to promote anthracycline resistance in breast cancer. In contrast, miR-200c raised the expression of PTEN, contributing to reverse drug resistance (48). Not only can the PTEN protein affect the PI3K-AKT pathway, but this pathway also accepts other regulations. For example, insulin-like growth factor-1 receptor (IGF-1R) is a tyrosine kinase receptor that can activate the expression of PI3K-AKT and prevent cell apoptosis. Zhang, H. et al. found that the downregulation of miR-520b expression can increase the expression of PI3K-AKT by activating IGF-1R so that tumor cells can acquire drug resistance (49). Similarly, the downregulation of miR-452 can also induce drug resistance through the IGF-1 signaling pathway (50). Other studies have shown that miR-7 can restrain the expression of epidermal growth factor receptor (EGFR), thereby regulating the PI3K-AKT pathway to enhance the sensitivity of cells to anthracycline drugs (51). miR-200c can enhance tumor cell resistance to anthracyclines by decreasing the expression of Friend of GATA2 (FOG2) protein (52). In addition to the above pathways, vascular endothelial growth factor A (VEGFA) and fibroblast growth factor 2 (FGF2) also target the PI3K-AKT pathway. miR-205 has also been shown to inhibit the synthesis of VEGFA and FGF2, causing damage to the pathway and tumor cell apoptosis (53).

The Notch gene, as a highly conserved gene, regulates cell proliferation, differentiation, and apoptosis (54, 55). It also plays an important role in the interaction of adjacent cells, which makes the Notch pathway a possible target for tumor treatment. Among published studies, miR-34a is closely related to the Notch pathway (56). The expression of homolog 1 declined after downregulating miR-34a. Cell drug resistance is reduced because of the inhibition of the Notch pathway, after upregulating miR-34a (57). Moreover, it has been reported that miR-34a can suppress tumor cell migration effectively through the Notch pathway (28).

Beyond the above pathways, the mitogen-activated protein kinase/the extracellular signal-related kinases (MAPK/ERK) pathway is also a potential direction that countributes to regulate the resistance to anthracyclines (58). The MAPK family are highly conserved serine/threonine protein kinases, a group of major signaling molecules in the process of signal transduction. Thus, it plays an important role in cancer development and disease occurrence (59). It has been reported that miR-302s inhibited the transcription of P-gp glycoprotein and resensitized the resistance of breast cancer cells to Adriamycin (ADR) to death by reducing the expression of mitogen-activated protein kinase/ERK kinase 1 (MEKK1) (21). Furthermore, P38 is an another important member of the MAPK family. miR-381 executes its function by inhibiting the expression of Fyn, a Src-family kinase (60) and cutting down the synthesis of P38 (61). This means that it may become a potential target for overcoming the resistance of anthracyclines in the future. Tyrosine 3-monooxygenase/tryptophan 5-monooxygenase activation protein zeta (YWHAZ), an antiapoptotic gene located downstream of miR-30c, can inhibit the expression of the P38 pathway (62). Other pathways have also been mentioned. For example, miR-140-5p was found to suppress the Wnt1 pathway, resulting in decline of anthracyclines resistance (63). Another miRNA, miR-148a, plays a role in tumor migration and erosion by inhibiting the expression of the Wnt-1 pathway (64). Lastly, miR-129-5p induced apoptosis of cancer cells by disrupting SOX2 expression (65).

### MiRNAs and the Cell Cycle

In addition to the function of apoptosis and the EMT process in the formation of drug resistance, abnormal cell cycle is also an important factor to consider. As we all know, cancer cells can produce infinite proliferation, so uncontrollable proliferation is a manifestation of cancer drug resistance. The cell cycle includes the division phase and the interphase. When the cell cycle is in the G0 phase, the cells stop dividing. Therefore, restraining the cancer cell cycle in the G0 phase is a potential way to overcome cancer drug resistance. Cyclin-dependent kinase (CDK), a regulator of the cell cycle, are divided into two categories based on their positive and negative effects (66). The positive regulation is *via* cyclin and the negative regulation is *via* cyclin-dependent protein kinase inhibitor (CKI) (67). The reduced expression of p27kip, a CKI regulated by miR-222, causing proliferation of tumor cells and reducing apoptosis. Based on the view of Wang, D.D. et al., the IC50 of tumor cells was increased after upregulating miR-222, which indicated that the increased resistance to anthracyclines (68). Additionly, miR-24 inhibited expression of p27kip (69, 70). It changed the chemosensitivity by regulating autophagy and tumor vascular survival. miR-574 has also been found to inhibit the expression of Smad4. It accelerates the G1-S phase of the cell cycle through regulating the Transforming Growth Factor-β (TGF-β), inducing cell growth and reducing the sensitivity of cells to anthracyclines (71). Other studies have discovered a negative feedback regulation of CDKs. miR-449 could inhibit cell cycle gene expression against drug resistance by reducing the synthesis of CDK2, E2F transcription factor 1 (E2F1), E2F transcription factor 3 (E2F3). Surprisingly, E2F1 regulates the expression of miR-449, forming a negative feedback loop (72). Furthermore, the aforementioned miR-122-5p can reduce the expression of drug resistance by targeting CDKs (27). A mircoRNA targeting cyclin was rarely discovered. The downregulation of miR-135b-5p was confirmed to promote the synthesis of Anterior Gradient 2 (AGR2), an enzyme that functions as a folding protein on the endoplasmic reticulum of tumor cells. It can mediate the expression of cyclin D1 and make cells sensitive to anthracyclines (73).

### MiRNAs and Epithelial-Mesenchymal Transition

EMT ensures the cells with the ability of transformation and invasion, therefore against cell senescence and apoptosis (74). EMT regulates the process of cell development, as well as participates in the process of tissue healing, cancer occurrence, and metastasis. So EMT is critical to the development of drug resistance in the treatment of breast cancer with anthracyclines (75). miR-93 was found to strengthen cell proliferation and reduce the sensitivity of tumor cells to the drug by inhibiting the PTEN pathway and promoting the occurrence of EMT (76). Du, F.Y. and his colleagues showed that the overexpression of miR-137 would be detrimental to the synthesis of dual-specifificity phosphatase 4 (DUSP4), which blocked the EMT process. Therefore, it implied that miR-137 has a great potential for sensitivity enhancement against anthracyclines resistance (77). In addition, studies have validated that miR-124 targets the expression of t signal transducer and activator of transcription 3 (STAT3), suppressing the activity of the hypoxia-inducible factor-1(HIF-1) pathway, resulting in the reversal of drug resistance (78). An intriguing discovery is the miR-448 positive feedback. It has been reported that the downregulation of miR-448 can promote the high expression of special AT-rich sequence-binding protein-1 (SATB1) sequence binding protein, sequentially activating the EMT process and the nuclear factor NF-κβ (79). Moreover, NF-κβ, when bonded to miR-448, inhibits the transcription of miR-448. This positive feedback phenomenon enhances the drug resistance of tumor cells. Vimentin and cadherin are important protein targets in the EMT process and are closely related to miRNAs. Zhou, Y. et al. found that miR-25 played a major role in the miR106b-25 cluster, which can reduce the expression of EP300, a transcriptional activator of E-cadherin. This change obstructed the occurrence of EMT and restored the chemosensitivity of tumor cells (80). Similarly, miR-181c renders tumor cells to regain sensitivity to anthracyclines by suppressing vimentin and N-cadherin (81). miR-489 performed its function of improving drug resistance of tumor cells by losing vimentin, but increasing the synthesis of E-cadherin, through reducing the synthesis of Smad3 (82, 83). Because there are many potential targets for a miRNA, a miRNA can also be regulated in many ways. As previously mentioned, it affects drug resistance through the PTEN-PI3K-AKT pathway and also can inhibit the EMT process by reducing the synthesis of E-calcein (48).

### MiRNAs and Other Mechanisms of Resistance

In addition to the above-mentioned key factors, exosomes have been discovered as having an emerging role. Exosomes encapsulates specific signaling molecules or biologically active molecules and can transmit from one cell to another, ventually, changing the biological activity of recipient cells (84). Tumor cells with anthracyclines resistance can deliver miRNA to sensitive tumor cells through exosomes so that sensitive cells can be transformed into resistant cells. Therefore, the emergence of drug resistance is also closely related to exosomes (85). From the prespective of Chen, W.X. et al., miR-222 and miR-100 are potential biomarkers that can be used to predic 4t drug resistance and prognosis. Moreover, upregulated miR-222 and miR-29 can be secreted from drug-resistant cells through exosomes and enter sensitive cells to infect them to endure anthracyclines (86). Among them, miR-222 was further studied to show that it can be transported from drug-resistant tumor cells to macrophages through exosomes. Finally, research showed its overall tumor cell resistance by causing inhibition of the PTEN pathway and remodeling of macrophages. Conversely, this mechanism can be used to improve medicinal properties to overcome drug resistance after anthracycline treatment of breast cancer. For example, drug-resistant cells combined with integrin and dvβ3 can deliver miR-159 through exosomes to inhibit tumor cell proliferation (87).

In addition, some miRNAs are associated with the anthracycline resistance of breast cancer cells through their unique effects. For example, miR-548p can elevate the synthesis of phenazine biosynthesis-like domain-containing protein (PBLD),which was identified as a tumor suppressor. Therefore, the overexpression of miR-548p expression restricted tumor growth and proliferation (88). miR-770 proomotes the polarization of macrophages by inhibiting the Stathmin1 (STMN1) protein, indicating that miR-770 controls the tumor microenvironment, which is conducive to reducing the drug resistance of tumor cells (89). miR-548c-3p and miR-1236-3p moderated drug resistance by inhibiting DNA damage and repair (52, 90). The downregulation of miR-149 contributes to the activation of heparinase by increasing the expression of GlcNAc N-deacetylase/N-sulfotransferase-1 (NDST1) and inducing drug resistance (91). In addition, miR-3609 causes tumor cells to become sensitive to anthracyclines through promoting the synthesis of Programmed death-ligand 1 (PDL1) (92). Meanwhile, Drug metabolizing enzymes and transporters can be inhibited by miR-148 and miR-152 to reduce the drug resistance of tumor cells (64). miR-133a plays its role in enhancing cell drug sensitivity by targeting ferritin light chain (FTL) protein (93). It has been shown that the decreasing expression of FBXW7, a tumor suppressor gene regulated by miR-188-5p, leads to the emergence of drug resistance (94).

### MiRNAs and Adverse Effects

**Figure 1:**
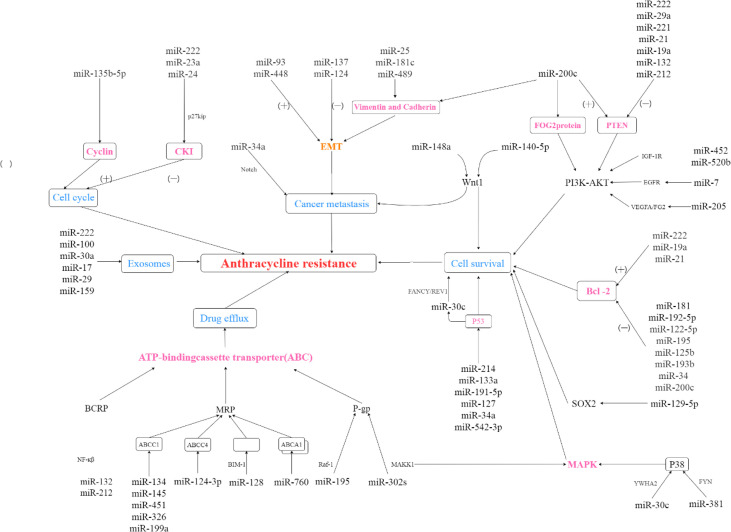
The role of miRNAs in anthracyclines resistance. (+): microRNA promotes expression of dowmstream protein; (-): microRNA suppresses expression of dowmstream protein.

In the process of applying anthracyclines to treat breast cancer patients, many adverse reactions often emerge. Among them, cardiovascular adverse reactions are the most prominent (95). Studies have shown that when using anthracycline chemotherapy regimens, the occurrence of cardiovascular events increases the risk of patient death (96). Therefore, urgent attention is needed on the occurrence of cardiovascular adverse events clinically. Appropriate stratification of risk factors and early detection is extremely meaningful for the survival and prognosis of breast cancer patients. Thus, a large number of miRNA related research is ongoing. miRNAs clearly show an objective quantitative or expression-intensity correlation with the occurrence of adverse reactions, which may help avoid or reduce the harm caused by adverse reactions to patients. It has guiding significance in improving the prognosis. Furthermore, X-ray photography, a commonly used method in clinical diagnosis, has the unavoidable disadvantage of discomfort, overdiagnosis, and false positives. **Figure 1** shows that the different roles of above mentioned miRNAs plays in anthracyclines resistance. As a detection method of peripheral blood indicators, miRNA is more nontoxic, convenient (97).

Qin, X. et al. (98). showed that anthracyclines induce myocardium damage in three possible ways: Firstly, it increased free radicals can cause tissue lipid oxidation leading to destruction of sarcomeres and causing autophagy and apoptosis of cardiomyocytes. The second mechanism is that it renders the death of cardiomyocytes by influencing topoisomerase II and opening the double strands of DNA of cardiomyocytes. Lastly, anthracyclines can damage myocardial fibers by inhibiting the ErbB-2 pathway (99). Anthacyclines induce early toxicity particularly in the left ventricle. The process of reconstruction is closely related to the reactivation of embryonic genes (100). miRNA can contribute to the cardiotoxicity occurrence (101).

The lethal-7 (let-7) family, a member of miRNAs, is mentioned extensivly. Upregulation of let-7a can predict the occurrence of cardiotoxicity (102). The downregulation of the expression of let-7f, a family member of let-7, signified the appearance of cardiac dysfunction because of its good correlation with N-terminal pro-B type natriuretic peptide (NT-proBNP) (98, 103). In addition to the let-7 family, miR-1 is another important biomarker, and has also been used as a clinical indicator. Expression of miR-1 implies the occurrence of arrhythmia to heart failure through a good response with left ventricular ejection fraction (LVEF) (98). Rigaud. et al. further explored its mechanism by demonstrating that miR-1 has a direct connection with the inhibition of antioxidant genes, leading to oxidative stress, promoting cardiomyocyte apoptosis, and myocardial damage (104). In another study, it was also found that miR-1 expression was increased in patients with adverse cardiotoxic reactions, possibly indicating that miR-1 was released by necrotic cardiomyocytes. miR-20a, miR-210, miR-34a, miR-126, and miR-130a are also have great potential. There is evidence that miR-20a is a dependable predictor of the occurrence of cardiotoxicity *via* the mechanism of activating angiogenesis and abnormal tumor vascular development. Another miRNA that plays a role in the development of chemoresistance, metastasis, proliferation, and self-renewal of tumor cells from hypoxic conditions is miR-210 (103). miR-34a-5p was also significantly increased after treatment with anthracyclines, which induces DNA breakage and P53 activation (105). MiR-126 also has the potential to be used to predict cardiotoxicity because it was validated to protect cardiomyocyte from apoptosis with its elevated expression (106–108). Recent findings have suggested that β-adrenergic pathway can modulate the contractile function in the heart by stimulating guanylyl nucleotide binding proteins, including adenylyl cyclase,cyclic adenosine monophosphate (cAMP), and so on (109–111). miR-30 affects the activation of the elements of the contraction coupling system by targeting the expression of β-adrenergic receptors. It was also concluded that miR-30 is a cardioprotective biomarker to monitor calcium overload and myocardial damage (112, 113).

In addition, another common adverse effect is liver metastasis. Studies have shown that miR-1-3p can mediate the occurrence of liver injury during the application of anthracyclines (4). Meanwhile, miRNA also plays a role in regulating the metastasis of breast cancer. miR-222 can promote the EMT process through the the mitogen-activated protein kinase (RAS-RAF-MEK-ERK) pathway, inducing breast cancer to be more aggressive and metastatic (68). Moreover, Deng, Z. et al. found that myelosuppressive cells (MDSC) can release miR-126a through exosomes to generate T helper 2 (Th2) cells with Interleukin-13 (IL-13) positive after Dox treatment. It promoted tumor angiogenesis, and ultimately led to breast cancer metastasis to the lungs (5). This also shows that miR-126a has the potential to predict breast cancer metastasis. **Table 1** presents the link of several miRNAs involved in response to adverse effects of anthracyclines treatment in breast cancer.

**Table 1:** MicroRNAs involved in response to adverse effects linked to anthracyclines treatment in breast cancer.

microRNA expression	Adverse effect	Reference
let-7a(↑)	cardiotoxicity	(102)
let-7f(↓)	cardiac dysfunction	(98, 103)
miR-1(↑)	Arrhythmia, heart failure	(98, 103, 104)
miR-20a(↑)	cardiotoxicity	(103)
miR-210(↑)	tumor metastasis	(98, 103)
miR-34a-5p(↑)	P53 activation, cardiotoxicity	(105) (98)
miR-130a(↓)	myocardial damage	(98)
miR-1-3p(↑)	liver injury	(4)
miR-222(↑)	tumor metastasis	(117)
miR-126a(↑)	lung metastasis	(5)

↑, microRNA expression increasing; ↓, microRNA expression decline.

## Conclusions and Outlook

There are many mechanisms and signaling pathways involved in the miRNA implications in breast cancer patients being treated with anthracyclines, and there is an abundance of possible miRNAs involved in the diagnosis, prognosis, and treatment of breast cancer patients. The study of microRNAs in regulating the resistance of anthracyclines in the treatment of breast cancer signifies that people may be able to control various types of microRNAs to overcome the shortcomings of anthracyclines application. MicroRNAs may be the key to the selection and development of personalized and safe anticancer drugs in the future. Moreover, as mentioned above, microRNAs can be used as a more accurate and safe early prediction tool, and has the potential as prognostic markers to be used in the clinic. Therefore, to overcome the challenge of providing individualized medicine in a complex disease, especially breast cancer, it is essential that more understanding of the biological effects of the various miRNAs will guide the possible direction of future academic interests.

Moreover, there are many challenges in current researches. Firstly, many groups of miRNAs involving breast cancer were found by metabonomics methods (9, 114, 115). To a certain extent, these results only found a simple quantitative relationship between them. But do not provide adequate epidemiological information, such as age, the course of disease, the breast cancer type and stage, complications, the dosage and dosing interval of anthracyclines administration and so on. More importantly, most studies do not consider whether drug interactions, especially some cardioprotective drugs, have influence on the outcomes. Whether this relationship could be used as a reliable early detection indicator for the clinic is doubtful. Therefore, more effort should be invested into a more deeper study. Lastly, not only are the traditional techniques of miRNA detection complex and require special laboratory skills, but they can also generate false-positives during the amplification process (116). The optimization of methods for quantification and visualization of abnormal miRNA expression are needed for early clinical diagnosis. A more accurate and economical detection method is an absolutely need in the future.

## Data Availability Statement

The original contributions presented in the study are included in the article/supplementary material. Further inquiries can be directed to the corresponding authors.

## Author Contributions

ZS and WZ: conceptualization, interpretation of data, and writing original draft. SL and YW: review, interpretation of data, and editing. WZ, GZ and YZ: conceptualization, writing original draft, review, editing, and supervision. All authors contributed to the article and approved the submitted version.

## Funding

This study was supported by the Baoan District Medical and Health Basic Research Project (2021JD054).

## Conflict of Interest

The authors declare that the research was conducted in the absence of any commercial or financial relationships that could be construed as a potential conflict of interest.

## Publisher’s Note

All claims expressed in this article are solely those of the authors and do not necessarily represent those of their affiliated organizations, or those of the publisher, the editors and the reviewers. Any product that may be evaluated in this article, or claim that may be made by its manufacturer, is not guaranteed or endorsed by the publisher.

## References

[1] Momparler RL Karon M Siegel SE Avila F . Effect of Adriamycin on DNA, RNA, and Protein Synthesis in Cell-Free Systems and Lntactcells1. Cancer Res (1976) 36(8):2891–5. 1277199

[2] Pommier Y Leo E Zhang H Marchand C . DNA Topoisomerases and Their Poisoning by Anticancer and Antibacterial Drugs. Chem Biol (2010) 17(5):421–33. doi: 10.1016/j.chembiol.2010.04.012 PMC7316379 20534341

[3] Vejpongsa P Yeh ET . Prevention of Anthracycline-Induced Cardiotoxicity: Challenges and Opportunities. J Am Coll Cardiol (2014) 64(9):938–45. doi: 10.1016/j.jacc.2014.06.1167 25169180

[4] Zhang Y Wang D Shen D Luo Y Che YQ . Identification of Exosomal miRNAs Associated With the Anthracycline-Induced Liver Injury in Postoperative Breast Cancer Patients by Small RNA Sequencing. PeerJ (2020) 8:e9021. doi: 10.7717/peerj.9021 32355577 PMC7185038

[5] Deng Z Rong Y Teng Y Zhuang X Samykutty A Mu J . Exosomes miR-126a Released From MDSC Induced by DOX Treatment Promotes Lung Metastasis. Oncogene (2017) 36(5):639–51. doi: 10.1038/onc.2016.229 PMC5419051 27345402

[6] Anglicheau D Muthukumar T Suthanthiran M . MicroRNAs: Small RNAs With Big Effects. Transplantation (2010) 90(2):105–12. doi: 10.1097/TP.0b013e3181e913c2 PMC3094098 20574417

[7] McManus MT . MicroRNAs and Cancer. Semin Cancer Biol (2003) 13(4):253–8. doi: 10.1016/s1044-579x(03)00038-5 14563119

[8] Holohan C Van Schaeybroeck S Longley DB Johnston PG . Cancer Drug Resistance: An Evolving Paradigm. Nat Rev Cancer (2013) 13(10):714–26. doi: 10.1038/nrc3599 24060863

[9] Li XJ Zha QB Ren ZJ Tang JH Yao YF . Mechanisms of Breast Cancer Resistance to Anthracyclines or Taxanes: An Overview of the Proposed Roles of Noncoding RNA. Curr Opin Oncol (2015) 27(6):457–65. doi: 10.1097/CCO.0000000000000235 26371779

[10] Yadi W Shurui C Tong Z Suxian C Qing T Dongning H . Bioinformatic Analysis of Peripheral Blood miRNA of Breast Cancer Patients in Relation With Anthracycline Cardiotoxicity. BMC Cardiovasc Disord (2020) 20(1):43. doi: 10.1186/s12872-020-01346-y 32013934 PMC6998363

[11] Xie M Fu Z Cao J Liu Y Wu J Li Q . MicroRNA-132 and microRNA-212 Mediate Doxorubicin Resistance by Down-Regulating the PTEN-AKT/NF-κB Signaling Pathway in Breast Cancer. Biomed Pharmacother (2018) 102:286–94. doi: 10.1016/j.biopha.2018.03.088 29567542

[12] Turashvili G Lightbody ED Tyryshkin K SenGupta SK Elliott BE Madarnas Y . Novel Prognostic and Predictive microRNA Targets for Triple-Negative Breast Cancer. FASEB J (2018) 32(11):5937–54. doi: 10.1096/fj.201800120R 29812973

[13] Lv J Fu Z Shi M Xia K Ji C Xu P . Systematic Analysis of Gene Expression Pattern in has-miR-760 Overexpressed Resistance of the MCF-7 Human Breast Cancer Cell to Doxorubicin. Biomed Pharmacother (2015) 69:162–9. doi: 10.1016/j.biopha.2014.11.028 25661353

[14] Lu L Ju F Zhao H Ma X . MicroRNA-134 Modulates Resistance to Doxorubicin in Human Breast Cancer Cells by Downregulating ABCC1. Biotechnol Lett (2015) 37(12):2387–94. doi: 10.1007/s10529-015-1941-y 26318721

[15] Gao M Miao L Liu M Li C Yu C Yan H . miR-145 Sensitizes Breast Cancer to Doxorubicin by Targeting Multidrug Resistance-Associated Protein-1. Oncotarget (2016) 7(37):59714–26. doi: 10.18632/oncotarget.10845 PMC5312343 27487127

[16] Kovalchuk O Filkowski J Meservy J Ilnytskyy Y Tryndyak VP Chekhun VF . Involvement of microRNA-451 in Resistance of the MCF-7 Breast Cancer Cells to Chemotherapeutic Drug Doxorubicin. Mol Cancer Ther (2008) 7(7):2152–9. doi: 10.1158/1535-7163.Mct-08-0021 18645025

[17] Liang Z Wu H Xia J Li Y Zhang Y Huang K . Involvement of miR-326 in Chemotherapy Resistance of Breast Cancer Through Modulating Expression of Multidrug Resistance-Associated Protein 1. Biochem Pharmacol (2010) 79(6):817–24. doi: 10.1016/j.bcp.2009.10.017 19883630

[18] Chang L Hu Z Zhou Z Zhang H . Linc00518 Contributes to Multidrug Resistance Through Regulating the MiR-199a/MRP1 Axis in Breast Cancer. Cell Physiol Biochem (2018) 48(1):16–28. doi: 10.1159/000491659 30001527

[19] Hu D Li M Su J Miao K Qiu X . Dual-Targeting of miR-124-3p and ABCC4 Promotes Sensitivity to Adriamycin in Breast Cancer Cells. Genet Test Mol Biomarkers (2019) 23(3):156–65. doi: 10.1089/gtmb.2018.0259 30807260

[20] Pearce HL Winter MA Beck WT . Structural Characteristics of Compounds That Modulate P-Glycoprotein-Associated Multidrug Resistance. Adv Enzyme Regul (1990) 30:357–73. doi: 10.1016/0065-2571(90)90026-x 1976291

[21] Zhao L Wang Y Jiang L He M Bai X Yu L . MiR-302a/B/C/D Cooperatively Sensitizes Breast Cancer Cells to Adriamycin *via* Suppressing P-Glycoprotein(P-Gp) by Targeting MAP/ERK Kinase Kinase 1 (MEKK1). J Exp Clin Cancer Res (2016) 35:25. doi: 10.1186/s13046-016-0300-8 26842910 PMC4738800

[22] Yang G Wu D Zhu J Jiang O Shi Q Tian J . Upregulation of miR-195 Increases the Sensitivity of Breast Cancer Cells to Adriamycin Treatment Through Inhibition of Raf-1. Oncol Rep (2013) 30(2):877–89. doi: 10.3892/or.2013.2532 23760062

[23] He H Shao X Li Y Gihu R Xie H Zhou J . Targeting Signaling Pathway Networks in Several Malignant Tumors: Progresses and Challenges. Front Pharmacol (2021) 12:675675. doi: 10.3389/fphar.2021.675675 34135756 PMC8203325

[24] Xie X Hu Y Xu L Fu Y Tu J Zhao H . The Role of miR-125b-Mitochondria-Caspase-3 Pathway in Doxorubicin Resistance and Therapy in Human Breast Cancer. Tumour Biol (2015) 36(9):7185–94. doi: 10.1007/s13277-015-3438-7 25894378

[25] Zhu Y Wu J Li S Ma R Cao H Ji M . The Function Role of miR-181a in Chemosensitivity to Adriamycin by Targeting Bcl-2 in Low-Invasive Breast Cancer Cells. Cell Physiol (2013) 32(5):1225–37. doi: 10.1159/000354521 24335172

[26] Zhang Y He Y Lu LL Zhou ZY Wan NB Li GP . miRNA-192-5p Impacts the Sensitivity of Breast Cancer Cells to Doxorubicin *via* Targeting Peptidylprolyl Isomerase a. Kaohsiung J Med Sci (2019) 35(1):17–23. doi: 10.1002/kjm2.12004 30844143 PMC11900784

[27] Zhang W Jiang H Chen Y Ren F . Resveratrol Chemosensitizes Adriamycin-Resistant Breast Cancer Cells by Modulating miR-122-5p. J Cell Biochem (2019) 120(9):16283–92. doi: 10.1002/jcb.28910 31155753

[28] Deng X Cao M Zhang J Hu K Yin Z Zhou Z . Hyaluronic Acid-Chitosan Nanoparticles for Co-Delivery of MiR-34a and Doxorubicin in Therapy Against Triple Negative Breast Cancer. Biomaterials (2014) 35(14):4333–44. doi: 10.1016/j.biomaterials.2014.02.006 24565525

[29] Gioffré S Chiesa M Cardinale DM Ricci V Vavassori C Cipolla CM . Circulating MicroRNAs as Potential Predictors of Anthracycline-Induced Troponin Elevation in Breast Cancer Patients: Diverging Effects of Doxorubicin and Epirubicin. J Clin Med (2020) 9(5):1418. doi: 10.3390/jcm9051418 PMC7290665 32403263

[30] Long J Ji Z Jiang K Wang Z Meng G . miR-193b Modulates Resistance to Doxorubicin in Human Breast Cancer Cells by Downregulating MCL-1. BioMed Res Int (2015) 2015:373574. doi: 10.1155/2015/373574 26526790 PMC4615858

[31] Kopp F Oak PS Wagner E Roidl A . miR-200c Sensitizes Breast Cancer Cells to Doxorubicin Treatment by Decreasing TrkB and Bmi1 Expression. PloS One (2012) 7(11):e50469. doi: 10.1371/journal.pone.0050469 23209748 PMC3510180

[32] Dai H Xu LY Qian Q Zhu QW Chen WX . MicroRNA-222 Promotes Drug Resistance to Doxorubicin in Breast Cancer *via* Regulation of miR-222/Bim Pathway. Biosci Rep (2019) 39(7):BSR20190650. doi: 10.1042/BSR20190650 PMC6629945 31273056

[33] Chen GQ Zhao ZW Zhou HY Liu YJ Yang HJ . Systematic Analysis of microRNA Involved in Resistance of the MCF-7 Human Breast Cancer Cell to Doxorubicin. Med Oncol (Northwood London England) (2010) 27(2):406–15. doi: 10.1007/s12032-009-9225-9 19412672

[34] Zhang J Su B Gong C Xi Q Chao T . miR-214 Promotes Apoptosis and Sensitizes Breast Cancer Cells to Doxorubicin by Targeting the RFWD2-P53 Cascade. Biochem Biophys Res Commun (2016) 478(1):337–42. doi: 10.1016/j.bbrc.2016.07.054 27422604

[35] Yuan Y Yao YF Hu SN Gao J Zhang LL . MiR-133a Is Functionally Involved in Doxorubicin-Resistance in Breast Cancer Cells MCF-7 *via* Its Regulation of the Expression of Uncoupling Protein 2. PloS One (2015) 10(6):e0129843. doi: 10.1371/journal.pone.0129843 26107945 PMC4481265

[36] Baffy G Derdak Z Robson SC . Mitochondrial Recoupling: A Novel Therapeutic Strategy for Cancer? Br J Cancer (2011) 105(4):469–74. doi: 10.1038/bjc.2011.245 PMC3170958 21712825

[37] Sharma S Nagpal N Ghosh PC Kulshreshtha R . P53-miR-191-SOX4 Regulatory Loop Affects Apoptosis in Breast Cancer. RNA (New York NY) (2017) 23(8):1237–46. doi: 10.1261/rna.060657.117 PMC5513068 28450532

[38] Lin S Yu L Song X Bi J Jiang L Wang Y . Intrinsic Adriamycin Resistance in P53-Mutated Breast Cancer is Related to the miR-30c/FANCF/REV1-Mediated DNA Damage Response. Cell Death Dis (2019) 10(9):666. doi: 10.1038/s41419-019-1871-z 31511498 PMC6739306

[39] Wang S Zhang J Wang Y Chen M . Hyaluronic Acid-Coated PEI-PLGA Nanoparticles Mediated Co-Delivery of Doxorubicin and miR-542-3p for Triple Negative Breast Cancer Therapy. Nanomed Nanotechnol Biol Med (2016) 12(2):411–20. doi: 10.1016/j.nano.2015.09.014 26711968

[40] Gu F Ma Y Zhang J Qin F Fu L . Function of Slit/Robo Signaling in Breast Cancer. Front Med (2015) 9(4):431–6. doi: 10.1007/s11684-015-0416-9 26542734

[41] Goncalves MD Hopkins BD Cantley LC . Phosphatidylinositol 3-Kinase, Growth Disorders, and Cancer. N Engl J Med (2018) 379(21):2052–62. doi: 10.1056/NEJMra1704560 30462943

[42] Wang DD Yang SJ Chen X Shen HY Luo LJ Zhang XH . miR-222 Induces Adriamycin Resistance in Breast Cancer Through PTEN/Akt/p27(kip1) Pathway. Tumour Biol (2016) 37(11):15315–24. doi: 10.1007/s13277-016-5341-2 27699665

[43] Shen H Wang D Li L Yang S Chen X Zhou S . MiR-222 Promotes Drug-Resistance of Breast Cancer Cells to Adriamycin *via* Modulation of PTEN/Akt/FOXO1 Pathway. Gene (2017) 596:110–8. doi: 10.1016/j.gene.2016.10.016 27746366

[44] Zhong S Li W Chen Z Xu J Zhao J . MiR-222 and miR-29a Contribute to the Drug-Resistance of Breast Cancer Cells. Gene (2013) 531(1):8–14. doi: 10.1016/j.gene.2013.08.062 23994196

[45] Wang ZX Lu BB Wang H Cheng ZX Yin YM . MicroRNA-21 Modulates Chemosensitivity of Breast Cancer Cells to Doxorubicin by Targeting PTEN. Arch Med Res (2011) 42(4):281–90. doi: 10.1016/j.arcmed.2011.06.008 21820606

[46] Rui M Qu Y Gao T Ge Y Feng C Xu X . Simultaneous Delivery of Anti-Mir21 With Doxorubicin Prodrug by Mimetic Lipoprotein Nanoparticles for Synergistic Effect Against Drug Resistance in Cancer Cells. Int J Nanomedicine (2017) 12:217–37. doi: 10.2147/ijn.S122171 PMC5221799 28115844

[47] Li Q Liu M Ma F Luo Y Cai R Wang L . Circulating miR-19a and miR-205 in Serum may Predict the Sensitivity of Luminal A Subtype of Breast Cancer Patients to Neoadjuvant Chemotherapy With Epirubicin Plus Paclitaxel. PloS One (2014) 9(8):e104870. doi: 10.1371/journal.pone.0104870 25137071 PMC4138038

[48] Chen Y Sun Y Chen L Xu X Zhang X Wang B . miRNA-200c Increases the Sensitivity of Breast Cancer Cells to Doxorubicin Through the Suppression of E-Cadherin-Mediated PTEN/Akt Signaling. Mol Med Rep (2013) 7(5):1579–84. doi: 10.3892/mmr.2013.1403 23546450

[49] Zhang H Zheng XD Zeng XH Li L Zhou Q . miR-520b Inhibits IGF-1R to Increase Doxorubicin Sensitivity and Promote Cell Apoptosis in Breast Cancer. Yakugaku Zasshi (2021) 141(3):415–26. doi: 10.1248/yakushi.20-00160 33116033

[50] Hu Q Gong JP Li J Zhong SL Chen WX Zhang JY . Down-Regulation of miRNA-452 is Associated With Adriamycin-Resistance in Breast Cancer Cells. Asian Pac J Cancer Prev (2014) 15(13):5137–42. doi: 10.7314/apjcp.2014.15.13.5137 25040964

[51] Huang Q Wu YY Xing SJ Yu ZW . Effect of miR-7 on Resistance of Breast Cancer Cells to Adriamycin *via* Regulating EGFR/PI3K Signaling Pathway. Eur Rev Med Pharmacol Sci (2019) 23(12):5285–92. doi: 10.26355/eurrev_201906_18195 31298380

[52] Tormo E Pineda B Serna E Guijarro A Ribas G Fores J . MicroRNA Profile in Response to Doxorubicin Treatment in Breast Cancer. J Cell Biochem (2015) 116(9):2061–73. doi: 10.1002/jcb.25162 25802200

[53] Hu Y Qiu Y Yagüe E Ji W Liu J Zhang J . miRNA-205 Targets VEGFA and FGF2 and Regulates Resistance to Chemotherapeutics in Breast Cancer. Cell Death Dis (2016) 7(6):e2291. doi: 10.1038/cddis.2016.194 27362808 PMC5108343

[54] Zhou B Lin W Long Y Yang Y Zhang H Wu K . Notch Signaling Pathway: Architecture, Disease, and Therapeutics. Signal Transduct Target Ther (2022) 7(1):95. doi: 10.1038/s41392-022-00934-y 35332121 PMC8948217

[55] Andersson ER Sandberg R Lendahl U . Notch Signaling: Simplicity in Design, Versatility in Function. Development (2011) 138(17):3593–612. doi: 10.1242/dev.063610 21828089

[56] Li WJ Wang Y Liu R Kasinski AL Shen H Slack FJ . MicroRNA-34a: Potent Tumor Suppressor, Cancer Stem Cell Inhibitor, and Potential Anticancer Therapeutic. Front Cell Dev Biol (2021) 9:640587. doi: 10.3389/fcell.2021.640587 33763422 PMC7982597

[57] Li XJ Ji MH Zhong SL Zha QB Xu JJ Zhao JH . MicroRNA-34a Modulates Chemosensitivity of Breast Cancer Cells to Adriamycin by Targeting Notch1. Arch Med Res (2012) 43(7):514–21. doi: 10.1016/j.arcmed.2012.09.007 23085450

[58] Salaroglio IC Mungo E Gazzano E Kopecka J Riganti C . ERK is a Pivotal Player of Chemo-Immune-Resistance in Cancer. Int J Mol Sci (2019) 20(10):2505. doi: 10.3390/ijms20102505 PMC6566596 31117237

[59] Sun Y Liu WZ Liu T Feng X Yang N Zhou HF . Signaling Pathway of MAPK/ERK in Cell Proliferation, Differentiation, Migration, Senescence and Apoptosis. J Recept Signal Transduct Res (2015) 35(6):600–4. doi: 10.3109/10799893.2015.1030412 26096166

[60] Kefalas P Brown TR Brickell PM . Signalling by the P60c-Src Family of Protein-Tyrosine Kinases. Int J Biochem Cell Biol (1995) 27(6):551–63. doi: 10.1016/1357-2725(95)00024-J 7545532

[61] Mi H Wang X Wang F Li L Zhu M Wang N . miR-381 Induces Sensitivity of Breast Cancer Cells to Doxorubicin by Inactivation of MAPK Signaling *via* FYN. Eur J Pharmacol (2018) 839:66–75. doi: 10.1016/j.ejphar.2018.09.024 30266665

[62] Fang Y Shen H Cao Y Li H Qin R Chen Q . Involvement of miR-30c in Resistance to Doxorubicin by Regulating YWHAZ in Breast Cancer Cells. Braz J Med Biol Res (2014) 47(1):60–9. doi: 10.1590/1414-431x20133324 PMC3932974 24519092

[63] Wu D Zhang J Lu Y Bo S Li L Wang L . miR-140-5p Inhibits the Proliferation and Enhances the Efficacy of Doxorubicin to Breast Cancer Stem Cells by Targeting Wnt1. Cancer Gene Ther (2019) 26(3-4):74–82. doi: 10.1038/s41417-018-0035-0 30032164

[64] Chen X Wang YW Gao P . SPIN1, Negatively Regulated by miR-148/152, Enhances Adriamycin Resistance *via* Upregulating Drug Metabolizing Enzymes and Transporter in Breast Cancer. J Exp Clin Cancer Res (2018) 37(1):100. doi: 10.1186/s13046-018-0748-9 29743122 PMC5944004

[65] Zeng H Wang L Wang J Chen T Li H Zhang K . microRNA-129-5p Suppresses Adriamycin Resistance in Breast Cancer by Targeting SOX2. Arch Biochem Biophys (2018) 651:52–60. doi: 10.1016/j.abb.2018.05.018 29802821

[66] Malumbres M Barbacid M . Cell Cycle, CDKs and Cancer: A Changing Paradigm. Nat Rev Cancer (2009) 9(3):153–66. doi: 10.1038/nrc2602 19238148

[67] Vermeulen K Berneman ZN Bockstaele DRV . Cell Cycle and Apoptosis. Cell Prolif (2003) 36(3):165–75. doi: 10.1046/j.1365-2184.2003.00267.x PMC6496173 12814432

[68] Wang DD Li J Sha HH Chen X Yang SJ Shen HY . miR-222 Confers the Resistance of Breast Cancer Cells to Adriamycin Through Suppression of P27(Kip1) Expression. Gene (2016) 590(1):44–50. doi: 10.1016/j.gene.2016.06.013 27282281

[69] Mao L Li J Chen WX Cai YQ Yu DD Zhong SL . Exosomes Decrease Sensitivity of Breast Cancer Cells to Adriamycin by Delivering microRNAs. Tumour (2016) 37(4):5247–56. doi: 10.1007/s13277-015-4402-2 26555545

[70] Giglio S Cirombella R Amodeo R Portaro L Lavra L Vecchione A . MicroRNA miR-24 Promotes Cell Proliferation by Targeting the CDKs Inhibitors p27Kip1 and P16ink4a. J Cell Physiol (2013) 228(10):2015–23. doi: 10.1002/jcp.24368 23553486

[71] Sun FD Wang PC Luan RL Zou SH Du X . MicroRNA-574 Enhances Doxorubicin Resistance Through Down-Regulating SMAD4 in Breast Cancer Cells. Eur Rev Med Pharmacol Sci (2018) 22(5):1342–50. doi: 10.26355/eurrev_201803_14476 29565492

[72] Tormo E Ballester S Adam-Artigues A Burgués O Alonso E Bermejo B . The miRNA-449 Family Mediates Doxorubicin Resistance in Triple-Negative Breast Cancer by Regulating Cell Cycle Factors. Sci Rep (2019) 9(1):5316. doi: 10.1038/s41598-019-41472-y 30926829 PMC6441107

[73] Zhang Y Xia F Zhang F Cui Y Wang Q Liu H . miR-135b-5p Enhances Doxorubicin-Sensitivity of Breast Cancer Cells Through Targeting Anterior Gradient 2. J Exp Clin Cancer Res (2019) 38(1):26. doi: 10.1186/s13046-019-1024-3 30665445 PMC6341729

[74] Pastushenko I Blanpain C . EMT Transition States During Tumor Progression and Metastasis. Trends Cell Biol (2019) 29(3):212–26. doi: 10.1016/j.tcb.2018.12.001 30594349

[75] Du B Shim JS . Targeting Epithelial-Mesenchymal Transition (EMT) to Overcome Drug Resistance in Cancer. Molecules (2016) 21(7):965. doi: 10.3390/molecules21070965 PMC6273543 27455225

[76] Chu S Liu G Xia P Chen G Shi F Yi T . miR-93 and PTEN: Key Regulators of Doxorubicin-Resistance and EMT in Breast Cancer. Oncol Rep (2017) 38(4):2401–7. doi: 10.3892/or.2017.5859 28765915

[77] Du F Yu L Wu Y Wang S Yao J Zheng X . miR-137 Alleviates Doxorubicin Resistance in Breast Cancer Through Inhibition of Epithelial-Mesenchymal Transition by Targeting DUSP4. Cell Death Dis (2019) 10(12):922. doi: 10.1038/s41419-019-2164-2 31801953 PMC6892819

[78] Liu C Xing H Guo C Yang Z Wang Y Wang Y . MiR-124 Reversed the Doxorubicin Resistance of Breast Cancer Stem Cells Through STAT3/HIF-1 Signaling Pathways. Cell Cycle (Georgetown Tex) (2019) 18(18):2215–27. doi: 10.1080/15384101.2019.1638182 PMC6738528 31286834

[79] Li QQ Chen ZQ Cao XX Xu JD Xu JW Chen YY . Involvement of NF-κb/miR-448 Regulatory Feedback Loop in Chemotherapy-Induced Epithelial-Mesenchymal Transition of Breast Cancer Cells. Cell Death Differ (2011) 18(1):16–25. doi: 10.1038/cdd.2010.103 20798686 PMC3131865

[80] Zhou Y Hu Y Yang M Jat P Li K Lombardo Y . The miR-106b~25 Cluster Promotes Bypass of Doxorubicin-Induced Senescence and Increase in Motility and Invasion by Targeting the E-Cadherin Transcriptional Activator EP300. Cell Death Differ (2014) 21(3):462–74. doi: 10.1038/cdd.2013.167 PMC3921594 24270410

[81] Han B Huang J Han Y Hao J Wu X Song H . The microRNA miR-181c Enhances Chemosensitivity and Reduces Chemoresistance in Breast Cancer Cells *via* Down-Regulating Osteopontin. Int J Biol Macromol (2019) 125:544–56. doi: 10.1016/j.ijbiomac.2018.12.075 30537505

[82] He DX Gu F Gao F Hao JJ Gong D Gu XT . Genome-Wide Profiles of Methylation, microRNAs, and Gene Expression in Chemoresistant Breast Cancer. Sci Rep (2016) 6:24706. doi: 10.1038/srep24706 27094684 PMC4837395

[83] Jiang L He D Yang D Chen Z Pan Q Mao A . MiR-489 Regulates Chemoresistance in Breast Cancer *via* Epithelial Mesenchymal Transition Pathway. FEBS Lett (2014) 588(11):2009–15. doi: 10.1016/j.febslet.2014.04.024 24786471

[84] Kalluri R LeBleu VS . The Biology, Function, and Biomedical Applications of Exosomes. Science (2020) 367(6478):3. doi: 10.1126/science.aau6977 PMC7717626 32029601

[85] Chen WX Liu XM Lv MM Chen L Zhao JH Zhong SL . Exosomes From Drug-Resistant Breast Cancer Cells Transmit Chemoresistance by a Horizontal Transfer of microRNAs. PloS One (2014) 9(4):e95240. doi: 10.1371/journal.pone.0095240 24740415 PMC3989268

[86] Zhong S Chen X Wang D Zhang X Shen H Yang S . MicroRNA Expression Profiles of Drug-Resistance Breast Cancer Cells and Their Exosomes. Oncotarget (2016) 7(15):19601–9. doi: 10.18632/oncotarget.7481 PMC4991404 26910922

[87] Gong C Tian J Wang Z Gao Y Wu X Ding X . Functional Exosome-Mediated Co-Delivery of Doxorubicin and Hydrophobically Modified microRNA 159 for Triple-Negative Breast Cancer Therapy. J Nanobiotechnology (2019) 17(1):93. doi: 10.1186/s12951-019-0526-7 31481080 PMC6721253

[88] Liang Y Song X Li Y Su P Han D Ma T . cirCKDM4C Suppresses Tumor Progression and Attenuates Doxorubicin Resistance by Regulating miR-548p/PBLD Axis in Breast Cancer. Oncogene (2019) 38(42):6850–66. doi: 10.1038/s41388-019-0926-z 31406252

[89] Li Y Liang Y Sang Y Song X Zhang H Liu Y . MiR-770 Suppresses the Chemo-Resistance and Metastasis of Triple Negative Breast Cancer *via* Direct Targeting of STMN1. Cell Death Dis (2018) 9(1):14. doi: 10.1038/s41419-017-0030-7 29323124 PMC5849036

[90] Hao J Du X Lv F Shi Q . Knockdown of Circ_0006528 Suppresses Cell Proliferation, Migration, Invasion, and Adriamycin Chemoresistance *via* Regulating the miR-1236-3p/CHD4 Axis in Breast Cancer. J Surg Res (2021) 260:104–15. doi: 10.1016/j.jss.2020.10.031 33333383

[91] He DX Gu XT Li YR Jiang L Jin J Ma X . Methylation-Regulated miR-149 Modulates Chemoresistance by Targeting GlcNAc N-Deacetylase/N-Sulfotransferase-1 in Human Breast Cancer. FEBS J (2014) 281(20):4718–30. doi: 10.1111/febs.13012 25156775

[92] Li D Wang X Yang M Kan Q Duan Z . Mir3609 Sensitizes Breast Cancer Cells to Adriamycin by Blocking the Programmed Death-Ligand 1 Immune Checkpoint. Exp Cell Res (2019) 380(1):20–8. doi: 10.1016/j.yexcr.2019.03.025 30904483

[93] Chekhun VF Lukyanova NY Burlaka CA Bezdenezhnykh NA Shpyleva SI Tryndyak VP . Iron Metabolism Disturbances in the MCF-7 Human Breast Cancer Cells With Acquired Resistance to Doxorubicin and Cisplatin. Int J Oncol (2013) 43(5):1481–6. doi: 10.3892/ijo.2013.2063 23969999

[94] Yi X Lou L Wang J Xiong J Zhou S . Honokiol Antagonizes Doxorubicin Resistance in Human Breast Cancer *via* miR-188-5p/FBXW7/c-Myc Pathway. Cancer Chemother Pharmacol (2021) 87(5):647–56. doi: 10.1007/s00280-021-04238-w 33544209

[95] Gianni L Herman EH Lipshultz SE Minotti G Sarvazyan N Sawyer DB . Anthracycline Cardiotoxicity: From Bench to Bedside. J Clin Oncol (2008) 26(22):3777–84. doi: 10.1200/JCO.2007.14.9401 PMC3018290 18669466

[96] Cardinale D Colombo A Bacchiani G Tedeschi I Meroni CA Veglia F . Early Detection of Anthracycline Cardiotoxicity and Improvement With Heart Failure Therapy. Circulation (2015) 131(22):1981–8. doi: 10.1161/CIRCULATIONAHA.114.013777 25948538

[97] Nassar FJ Chamandi G Tfaily MA Zgheib NK Nasr R . Peripheral Blood-Based Biopsy for Breast Cancer Risk Prediction and Early Detection. Front Med (Lausanne) (2020) 7:28. doi: 10.3389/fmed.2020.00028 32118013 PMC7026666

[98] Qin X Chang F Wang Z Jiang W . Correlation of Circulating Pro-Angiogenic miRNAs With Cardiotoxicity Induced by Epirubicin/Cyclophosphamide Followed by Docetaxel in Patients With Breast Cancer. Cancer Biomarkers section A Dis markers. (2018) 23(4):473–84. doi: 10.3233/cbm-181301 PMC13078584 30452398

[99] Sawyer DB Peng X Chen B Pentassuglia L Lim CC . Mechanisms of Anthracycline Cardiac Injury: Can We Identify Strategies for Cardioprotection? Prog Cardiovasc Dis (2010) 53(2):105–13. doi: 10.1016/j.pcad.2010.06.007 PMC2933091 20728697

[100] Roca-Alonso L Pellegrino L Castellano L Stebbing J . Breast Cancer Treatment and Adverse Cardiac Events: What are the Molecular Mechanisms? Cardiology (2012) 122(4):253–9. doi: 10.1159/000339858 22907032

[101] Fa HG Chang WG Zhang XJ Xiao DD Wang JX . Noncoding RNAs in Doxorubicin-Induced Cardiotoxicity and Their Potential as Biomarkers and Therapeutic Targets. Acta Pharmacol Sin (2021) 42(4):499–507. doi: 10.1038/s41401-020-0471-x 32694762 PMC8114921

[102] Todorova VK Makhoul I Wei J Klimberg VS . Circulating miRNA Profiles of Doxorubicin-Induced Cardiotoxicity in Breast Cancer Patients. Ann Clin Lab Sci (2017) 47(2):115–9. 28442511

[103] Pereira JD Tosatti JAG Simões R Luizon MR Gomes KB Alves MT . microRNAs Associated to Anthracycline-Induced Cardiotoxicity in Women With Breast Cancer: A Systematic Review and Pathway Analysis. Biomed Pharmacother (2020) 131:110709. doi: 10.1016/j.biopha.2020.110709 32937248

[104] Rigaud VO Ferreira LR Ayub-Ferreira SM Ávila MS Brandão SM Cruz FD . Circulating miR-1 as a Potential Biomarker of Doxorubicin-Induced Cardiotoxicity in Breast Cancer Patients. Oncotarget (2017) 8(4):6994–7002. doi: 10.18632/oncotarget.14355 28052002 PMC5351685

[105] Frères P Bouznad N Servais L Josse C Wenric S Poncin A . Variations of Circulating Cardiac Biomarkers During and After Anthracycline-Containing Chemotherapy in Breast Cancer Patients. BMC Cancer (2018) 18(1):102. doi: 10.1186/s12885-018-4015-4 29378531 PMC5789542

[106] An Q Han C Zhou Y Li F Li D Zhang X . Matrine Induces Cell Cycle Arrest and Apoptosis With Recovery of the Expression of miR-126 in the A549 non-Small Cell Lung Cancer Cell Line. Mol Med Rep (2016) 14(5):4042–8. doi: 10.3892/mmr.2016.5753 PMC5101874 27665734

[107] Zhu Z Li X Dong H Ke S Zheng WH . Let-7f and miRNA-126 Correlate With Reduced Cardiotoxicity Risk in Triple-Negative Breast Cancer Patients Who Underwent Neoadjuvant Chemotherapy. Int J Clin Exp Pathol (2018) 11(10):4987–95. PMC6962922 31949575

[108] Luo Q Guo D Liu G Chen G Hang M Jin M . Exosomes From MiR-126-Overexpressing Adscs Are Therapeutic in Relieving Acute Myocardial Ischaemic Injury. Cell Physiol Biochem (2017) 44(6):2105–16. doi: 10.1159/000485949 29241208

[109] Xiao RP . β-Adrenergic Signaling in the Heart: Dual Coupling of the β2-Adrenergic Receptor to Gs and Gi Proteins. Sci STKE (2001) 104:re15. doi: 10.1126/stke.2001.104.re15 11604549

[110] Yang J Liu Y Fan X Li Z Cheng Y . A Pathway and Network Review on Beta-Adrenoceptor Signaling and Beta Blockers in Cardiac Remodeling. Heart Fail Rev (2014) 19(6):799–814. doi: 10.1007/s10741-013-9417-4 24366330

[111] Najafi A Sequeira V Kuster DW van der Velden J . Beta-Adrenergic Receptor Signalling and its Functional Consequences in the Diseased Heart. Eur J Clin Invest (2016) 46(4):362–74. doi: 10.1111/eci.12598 26842371

[112] Saddic LA Muehlschlegel JD . Sarco"MiR" Friend or Foe: A Perspective on the Mechanisms of Doxorubicin-Induced Cardiomyopathy. Ann Trans Med (2016) 4(10):203. doi: 10.21037/atm.2016.05.30 PMC4885903 27294099

[113] Roca-Alonso L Castellano L Mills A Dabrowska AF Sikkel MB Pellegrino L . Myocardial MiR-30 Downregulation Triggered by Doxorubicin Drives Alterations in Beta-Adrenergic Signaling and Enhances Apoptosis. Cell Death Dis (2015) 6:e1754. doi: 10.1038/cddis.2015.89 25950484 PMC4669718

[114] Bertoli G Cava C Castiglioni I . MicroRNAs: New Biomarkers for Diagnosis, Prognosis, Therapy Prediction and Therapeutic Tools for Breast Cancer. Theranostics (2015) 5(10):1122–43. doi: 10.7150/thno.11543 PMC4508501 26199650

[115] Kahraman M Roske A Laufer T Fehlmann T Backes C Kern F . MicroRNA in Diagnosis and Therapy Monitoring of Early-Stage Triple-Negative Breast Cancer. Sci Rep (2018) 8(1):11584. doi: 10.1038/s41598-018-29917-2 30072748 PMC6072710

[116] Ye J Xu M Tian X Cai S Zeng S . Research Advances in the Detection of miRNA. J Pharm Anal (2019) 9(4):217–26. doi: 10.1016/j.jpha.2019.05.004 PMC6702429 31452959

[117] Chen WX Wang DD Zhu B Zhu YZ Zheng L Feng ZQ . Exosomal miR-222 From Adriamycin-Resistant MCF-7 Breast Cancer Cells Promote Macrophages M2 Polarization *via* PTEN/Akt to Induce Tumor Progression. Aging (2021) 13(7):10415–30. doi: 10.18632/aging.202802 PMC8064228 33752173

